# Postpartum questionnaire survey of women who tested negative in a non-invasive prenatal testing: examining negative emotions towards the test

**DOI:** 10.1038/s10038-020-00879-6

**Published:** 2020-12-03

**Authors:** Tatsuko Hirose, Nahoko Shirato, Mikiko Izumi, Keiko Miyagami, Akihiko Sekizawa

**Affiliations:** 1 Clinical Genetics Medical Center, Showa University Hospital, Tokyo, Japan; 2 Department of Obstetrics and Gynecology, Showa University School of Medicine, Tokyo, Japan

**Keywords:** Medical ethics, Human behaviour

## Abstract

Non-invasive prenatal testing (NIPT) is used worldwide to screen for fetal aneuploidy. Although previous studies on the psychosocial aspects of NIPT have focused on satisfaction regarding the test, we surveyed women who experienced negative emotions after receiving NIPT. From January 2018 to March 2019, we surveyed pregnant women whose NIPT results were negative, one year after the test. Of the 526 respondents, 35 (6.7%) regretted receiving NIPT and blamed themselves for taking it. We assigned this 6.7% of respondents to the negative emotion group. Although, 76.5% of the participants in the negative emotion group reported they would like to take NIPT for their next pregnancy, it was significantly lower as compared to the control group (92%). Furthermore, 31.9% of respondents in the control group reported that they would recommend similar tests to their relatives and friends. Conversely, in the negative emotion group, this proportion was lower at 17.1%. This suggests that guilt over testing may be meaningful. Thus, this study showed that some NIPT examinees regretted taking the test and blamed themselves. Respondents reported experiencing stress, anxiety, and depression even before NIPT affirming that it is important to address pregnant women’s psychosocial status during pre-test genetic counseling.

## Introduction

More than seven years have passed since non-invasive prenatal testing (NIPT) started in Japan. As of 2018, there were 5161 hospitals and clinics with Obstetrics and Gynecology departments in Japan [1]. As of March 2019, only 109 facilities (2.1%) performed NIPT [2]. The Japan NIPT Consortium has performed NIPT clinical studies since 2013. Eighty-four institutions were included in the clinical study, and more than 75,000 NIPTs were conducted in those institutions from April 2013 to March 2019 [3].

In Japan, genetic counseling (GC) is always required when receiving NIPT. A questionnaire survey of women with negative NIPT results was conducted one year after the test to confirm newborns’ prognoses [4]. Notably, more than 90% of pregnant women answered that GC before NIPT is needed and about 90% of women with negative NIPT results were satisfied with the test [4]. In addition, we previously investigated the psychological evaluation of pregnant women and their partners and their satisfaction with NIPT. Specifically, we performed a stress assessment using the Visual Analogue Scale immediately after the test and one year later [5]. Results showed that women’s stress was significantly reduced one year after the test [5]. In contrast, Lewis and colleagues showed that some pregnant women who received NIPT had ambivalent feelings owing to the long waiting time to receive the examination and receive the results [6]. In addition, Yotsumoto and colleagues reported in a questionnaire survey that NIPT-negative women experienced several ambivalent feelings one year later, including “Options in the case of a positive result”, “Guilt towards the child”, “Criticisms on NIPT from others”, “Denial of disabled people”, and “How to tell the child” [7].

While these studies primarily assessed the satisfaction of NIPT-negative pregnant women, testing can lead to negative emotions such as regret. However, few studies focused on these women’s demographics and background characteristics. Therefore, 1 year after undergoing the test, we surveyed women who had negative NIPT results and displayed negative emotions. Then, we clarified women’s demographics and background characteristics, such as age and parity, and examined their evaluation of NIPT and GC.

## Materials and methods

When administering NIPT at our hospital, pregnant women and their partners receive GC from a clinical geneticist or a certified genetic counselor for 45–60 min. Results will be reported to pregnant women at GC 1–2 weeks later. If the result is negative, pregnant women continue typical pregnancy management.

In our hospital, since the beginning of NIPT, we have been conducting a questionnaire survey for women who were NIPT-negative one year after receiving NIPT. This is mainly done to confirm children’s outcomes and women’s impressions of NIPT. In this study, to examine the demographics and background characteristics of pregnant women with negative emotions, the contents of the questionnaire were reexamined.

**Fig. 1:**
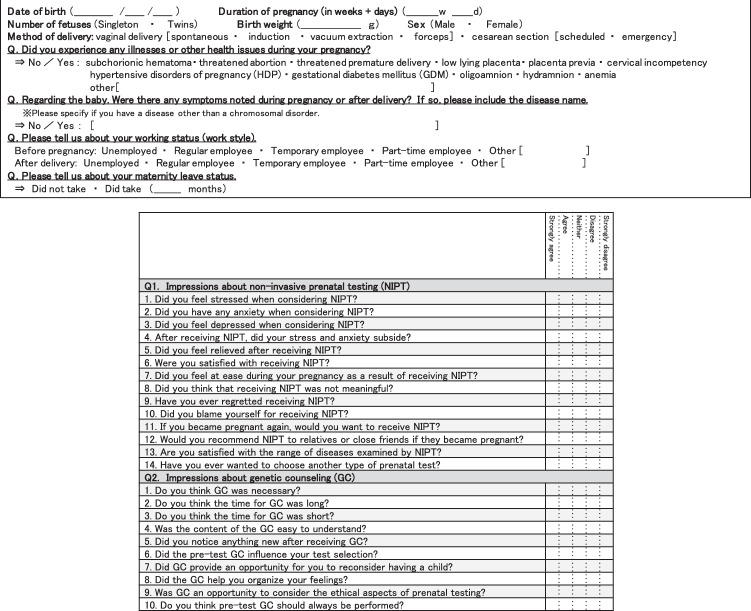
(1) Questionnaire 1 year after the NIPT examination (Part 1). Children’s outcome was answered by self-report. (2) Questionnaire one year after the NIPT examination (Part 2). We asked women about two types of items. Answers were rated on a five-point Likert scale from “strongly agree” to “strongly disagree”

The questionnaire included questions about child outcomes and women’s impressions of NIPT and GC. Responses were measured on a five-point Likert scale, ranging from “strongly agree” to “strongly disagree” (Fig. 1). The questionnaire was an anonymous, self-administered survey. By answering the questionnaire, the subject was considered to have agreed to the study. This study was conducted after being reviewed by the ethics committee of our hospital (no. 1580).

**Fig. 2:**
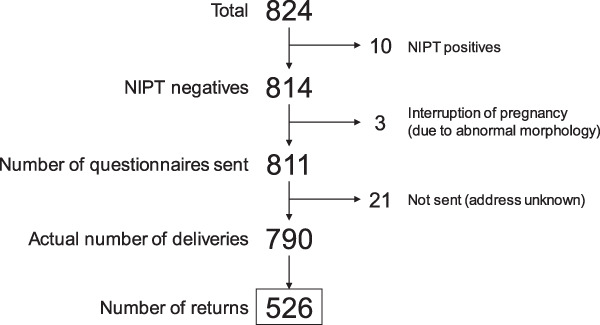
Number of questionnaires returned. A total of 526 (66.6%) women who were sent the questionnaire responded

Since 2013, our hospital has been participating in clinical study on NIPT conducted by the Japan NIPT Consortium [8], and the staff has performed an average of 708.7 ± 64.4 tests annually. The rate of positive NIPT results was 1.9%. In this study, from January 2018 to March 2019, we sent a questionnaire survey to NIPT-negative women who had received NIPT at our hospital. For the analysis, we used only the questionnaires that were answered by the end of March 2020. The number of NIPT examinees during the period of the study was 824; 10 received positive NIPT results and the rest received a negative NIPT result. Of those with negative NIPT results, a questionnaire was sent to 811 candidates one year after they took NIPT; we excluded three women who presented with fetal malformations during pregnancy and decided to terminate the pregnancy. Furthermore, we excluded 21 questionnaires that were deemed invalidated owing to mailing errors. Of these 790 questionnaires, 526 (66.6%) valid ones were returned (Fig. 2). Women’s age, gestational weeks of the examination, presence or absence of assisted reproductive technology, and previous history of pregnancy were confirmed in their medical records.

Questions 9 and 10 in Introduction section of the questionnaire were set up to assess the presence or absence of negative emotions. Those who replied, “strongly agree/agree” to at least one of the questions were classified as the “negative emotion group.” Those who replied, “disagree/strongly disagree” to at least one of the questions were classified as the control group. Background and impressions of NIPT and GC were compared between these two groups. Wilcoxon and chi-squared tests were used for statistical analysis, which were performed with JMP Pro 15 (SAS institute, Cary, NC, USA).

## Results

Among all respondents, 35 (negative emotion group: 6.7%) answered “strongly agree/agree” to at least one of the negative emotion items (questions 9 and 10 of section Q1). In contrast, 484 (control group: 92.0%) replied, “disagree/ strongly disagree” to at least one of the items. Seven answered “neither” to both questions.

Table 1 shows the comparison between the two groups concerning women’s demographics and background characteristics. There were no significant differences between the groups in all items regarding age, pregnancy duration, birth weight, reason for examination, pregnancy method, pregnancy history, miscarriage history, presence or absence of congenital disease, presence or absence of pregnancy complications, and working status.

**Table 1:** Background comparisons

		Negative emotion group (*N* = 35)	Control group (*N* = 484)	*P* value
Age at the time of NIPT (year)^a^		38.7 ± 2.6 (34–45)	38.4 ± 2.8 (27–47)	0.7228
Pregnancy duration (week)^a^		39.0 ± 2.4 (28.29–42.14)	38.8 ± 1.7 (26.14–41.86)	0.1526
Infant weight (g)^a^		3056.9 ± 504.8 (1266–3960)	2976.2 ± 433.6 (768–4153)	0.1976
Reason for examination^b^ (df = 3)	Advanced age	35	464	1.0000
	Childbirth history	0	13	
	Ultrasound findings	0	2	
	Serum marker positive	0	5	
Pregnancy method^b^ (df = 2)	Naturally	15	276	0.1953
	Fertility treatments	13	122	
	Unknown	7	86	
Parity^b^ (df = 1)	0	22	262	0.3806
	1 or more	13	222	
Miscarriage history^b^ (df = 1)	Yes	11	160	1.0000
	No	24	324	
Congenital disease^b^ (df = 2)	Yes	2	20	0.7574
	No	33	459	
	Unknown	0	5	
Pregnancy complications^b^ (df = 2)	Yes	16	212	0.8927
	No	19	258	
	Unknown	0	14	
Working status^b^ <before pregnancy> (df = 1)	Regular	20	302	0.5898
	Irregular or None	15	182	
Working status^b^ <after delivery> (df = 1)	Regular	17	265	0.4881
	Irregular or None	18	219	

The backgrounds of women in each group were compared

In the Infant weight, 23 of the control groups were twins and were excluded. Since some people did not indicate the Infant weight, the negative emotion group was 33 and the control group was 445

^a^Wilcoxon test (df = 1)

^b^Chi-square test

Next, we examined whether there were any differences between groups concerning women’s impressions with NIPT (Table 2). Regarding respondents’ mental state when receiving NIPT, we established the following questions: “Did you feel stressed when considering NIPT?” (Q1-1), “Did you have any anxiety when considering NIPT?” (Q1-2), and “Did you feel depressed when considering NIPT?” (Q1-3). The negative emotion group was significantly more likely to agree or strongly agree with these questions than was the control group (all ps < 0.001).

**Table 2:** Q1 comparisons

		Negative emotion group	Control group	*P* value
Q1-1.	Did you feel stressed when considering NIPT?	33(94.3)	228(47.4)	<0.0001^**^
Q1-2.	Did you have any anxiety when considering NIPT?	35(100)	335(69.2)	0.0001^*^
Q1-3.	Did you feel depressed when considering NIPT?	31(88.6)	172(35.6)	<0.0001^**^
Q1-4.	After receiving NIPT, did your stress and anxiety subside?	30(85.7)	435(90.1)	0.2387
Q1-5.	Did you feel relieved after receiving NIPT?	24(68.6)	389(80.5)	0.0217^*^
Q1-6.	Were you satisfied with receiving NIPT?	30(85.7)	479(99.0)	1.0000
Q1-7.	Did you feel at ease during your pregnancy as a result of receiving NIPT?	32(91.4)	453(93.6)	1.0000
Q1-8.	Did you think that receiving NIPT was not meaningful?	2(5.7)	4(0.8)	0.0514
Q1-11.	If you became pregnant again, would you want to receive NIPT?	26(76.5)	446(92.1)	0.0020^*^
Q1-12.	Would you recommend NIPT to relatives or close friends if they became pregnant?	6(17.1)	154(31.9)	0.0362^*^
Q1-13.	Are you satisfied with the range of diseases examined by NIPT?	19(54.3)	297(61.4)	1.0000
Q1-14.	Have you ever wanted to choose another type of prenatal test?	3(8.6)	13(2.7)	0.0888

The number and percentage (%) of the respondents who answered each question as either “strongly agree/agree”

*P < 0.05, **P < 0.001

Further, those in the negative emotion group scored their mental state after the test as significantly lower than did those in the control group (*p* < 0.05). Only 76% of respondents in the negative emotion group reported they would want to receive NIPT for their next pregnancy, which was significantly as compared to the control group (*p* < 0.005). Moreover, 17.1% respondents in the negative emotion group reported they would recommend NIPT to others, whereas this proportion was 31.9% in the control group (*p* < 0.05).

Then, we examined whether there were any differences between groups concerning women’s impressions with GC (Table 3). Women in the negative emotion group were more likely to report that pre-test GC influenced their test selection than were women in the control group (*p* < 0.005). However, there was no significant difference in other items, and both groups considered that GC was necessary. Women in both groups also generally believed that GC should always be performed before NIPT.

**Table 3:** Q2 comparisons

		Negative emotion group	Control group	*P* value
Q2-1.	Do you think GC was necessary?	29(82.9)	427(90.7)	0.5528
Q2-2.	Do you think the time for GC was long?	4(11.4)	34(7.2)	0.5128
Q2-3.	Do you think the time for GC was short?	1(2.9)	25(5.3)	1.0000
Q2-4.	Was the content of the GC easy to understand?	32(91.4)	451(95.3)	1.0000
Q2-5.	Did you notice anything new after receiving GC?	24(68.6)	338(71.8)	1.0000
Q2-6.	Did the pre-test GC influence your test selection?	13(37.1)	90(19.0)	0.0022^*^
Q2-7.	Did GC provide an opportunity for you to reconsider having a child?	24(70.6)	272(57.5)	0.5005
Q2-8.	Did the GC help you organize your feelings?	26(74.3)	335(71.0)	1.0000
Q2-9.	Was GC an opportunity to consider the ethical aspects of prenatal testing?	27(77.1)	335(70.9)	0.7586
Q2-10.	Do you think pre-test GC should always be performed?	30(85.7)	382(80.8)	1.0000

The number and percentage (%) of the respondents who answered each question as either “strongly agree/agree”

*P < 0.05, **P < 0.001

## Discussion

Of all the women who received negative NIPT results, 6.7% regretted or blamed themselves for the exam. In a previous study, 5% of women with positive serum screening and amniocentesis results regretted having undergone serum screening [9]. Another previous survey used the Decision Regret Scale (DRS), as an indicator of regret after decision-making, with 200 patients who underwent NIPT. Twelve women (6%) had elevated DRS (higher regret) even when NIPT results were negative [10]. In sum, the proportion of respondents in the negative emotion group in this study mirrored that of other previous reports. Understanding that there are women who experience regret over prenatal testing, it is important to engage in daily GC.

Our analysis revealed that women’s anxiety, stress, and depressive tendencies before the test tended to be stronger in the negative emotion group than in the control group. Suzumori and colleagues reported that pregnant women who undergo NIPT have greater stress and anxiety than pregnant women who do not [11]. In addition, more than one-third of the pregnant women who had a negative NIPT result still experienced strong state anxiety (transient anxiety in each period) even after disclosure of their results [12]. In this study, we showed a relationship between regret after the test as well as anxiety and stress during the test. Our results suggest that it is necessary to assess the anxiety and stress levels of pregnant woman during GC before the test, and to listen to their reasons for feeling anxiety and stress.

Although the control group had a lower rate of anxiety and increased satisfaction after NIPT, the negative emotion group showed less change than did the control group. The rate of feeling relieved after receiving NIPT was significantly lower in the negative emotion group than in the control group. This may indicate that, at the time the test was taken, respondents were still unsure about their choice of test. We believe it is possible to diminish anxiety by devising an environment in which pregnant women can easily express their anxieties during GC.

Nearly one-third of the control group reported they would recommend NIPT to their relatives and friends, which was less than we expected but significantly higher in comparison to the negative emotion group. This may be influenced by cultural circumstances specific to Japan. According to the recommendation on maternal serum screening issued by the Health Science Council in 1999, obstetricians do not have to actively recommend this test to pregnant women or inform them of it [13]. In addition, the guidelines issued in 2013 by the Japan Society of Obstetrics and Gynecology state that “it is not necessary for obstetricians to inform pregnant women about NIPT” [14]. Under these circumstances, awareness of prenatal testing may not be generalized yet owing to insufficient information.

In addition, “In Japan, abortion is criminalized based on the values of the patriarchal system in place since the Meiji era, and punitive views still exist toward women who abort. They may be in an environment where it is difficult to talk to others about hesitation, worries and anxiety with regard to their pregnancy.” [15] The existence of such views in Japanese society is also reflected by the fact that the choice of undergoing prenatal testing is generally thought to imply a future abortion. Actually, Japanese press reports on prenatal genetic testing often introduce not only the number of tests but also the abortion rate [16]. Therefore, it may be easy to link feelings of guilt with the choices women make. These cultural implications mean pregnant women may hesitate to talk to their relatives and close friends about prenatal testing. Furthermore, it is likely that this social environment causes negative emotions such as stress for pregnant women, which was apparent in a small portion of our sample.

The negative emotion group tended to consider GC as an opportunity to think about having children and its ethical aspects. Pregnant women with negative emotion were significantly more likely to be influenced by GC concerning their choice of prenatal testing than were controls. Thus, pregnant women with negative emotions may regret their testing choices, as they may be anxious about specific ethical aspects. In contrast, even among pregnant women with negative emotions, more than 75% wanted to take NIPT for their next pregnancy. Although this proportion was lower in the control group, it suggests that women approved of NIPT, even though it was associated with negative emotions.

This study had some limitations. We only analyzed returned questionnaires. Many of these respondents may have had a relatively good impression of NIPT. In addition, we did not consider whether there were any underlying depressive disorders among respondents. Future researchers should more carefully evaluate pregnant women’s mental status during the pre-test GC. Furthermore, all women were recruited from one hospital; therefore, it is possible that different results would be obtained if similar surveys were conducted across Japan. The current results should thus be interpreted with caution.

In conclusion, 6.7% of NIPT-negative pregnant women regretted being tested or blamed themselves after giving birth. These women experienced considerable stress, anxiety, and depression even before the test. In addition, women who experienced strong anxiety, even after a negative test results, should consider receiving GC again during their pregnancy. Therefore, it is important to understand the psychosocial status of pregnant women during pre-test GC to ensure they are provided with the necessary psychological support.
